# Supplementary material for: Community Structure Diversity of Endophytic Fungi in Cissampelos pareira from Different Habitats and Their α-Glucosidase Inhibitory Activity
Source: J Fungi (Basel). 2025 Aug 22;11(9):615. doi: 10.3390/jof11090615 (PMC12470284; doi:10.3390/jof11090615)
Supplement: Supplementary file 1 [file jof-11-00615-s001.zip › Figure S2 Dilution curves of endophytic fungi from the 24 samples.pdf]

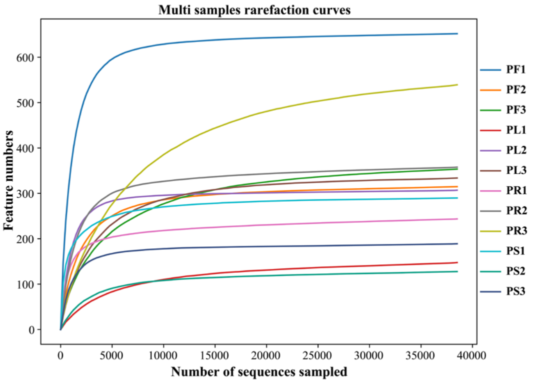

a

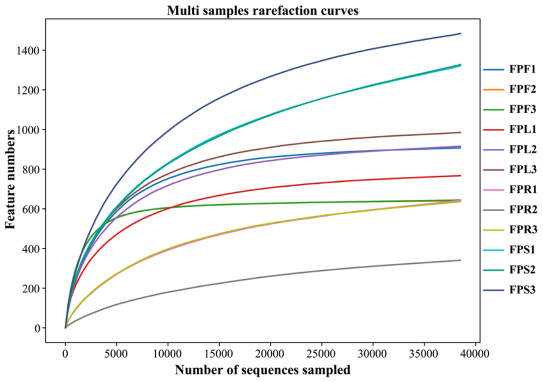

b

Figure S2. Dilution curves of endophytic fungi from the 24 samples. Dilution curves of endophytic fungi in potted *C. pareira* samples (**a**); Dilution curves of endophytic fungi in non-potted *C. pareira* samples (**b**). PR: potted *C. pareira* root, PS: potted *C. pareira* stem, PL: potted *C. pareira* leaf, PF: potted *C. pareira* flower, FPR: non-potted *C. pareira* root, FPS: non-potted *C. pareira* stem, FPL: non-potted *C. pareira* leaf, FPF: non-potted *C. pareira* flower. The numbers 1, 2, and 3 in the names are used to indicate that the same tissue sample is repeated three times.
