# Supplementary material for: Community Structure Diversity of Endophytic Fungi in Cissampelos pareira from Different Habitats and Their α-Glucosidase Inhibitory Activity
Source: J Fungi (Basel). 2025 Aug 22;11(9):615. doi: 10.3390/jof11090615 (PMC12470284; doi:10.3390/jof11090615)
Supplement: Supplementary file 1 [file jof-11-00615-s001.zip › Figure S5 Colonies of some endophytic fungi isolated from C. pareira on PDA cultured for 6 days.pdf]

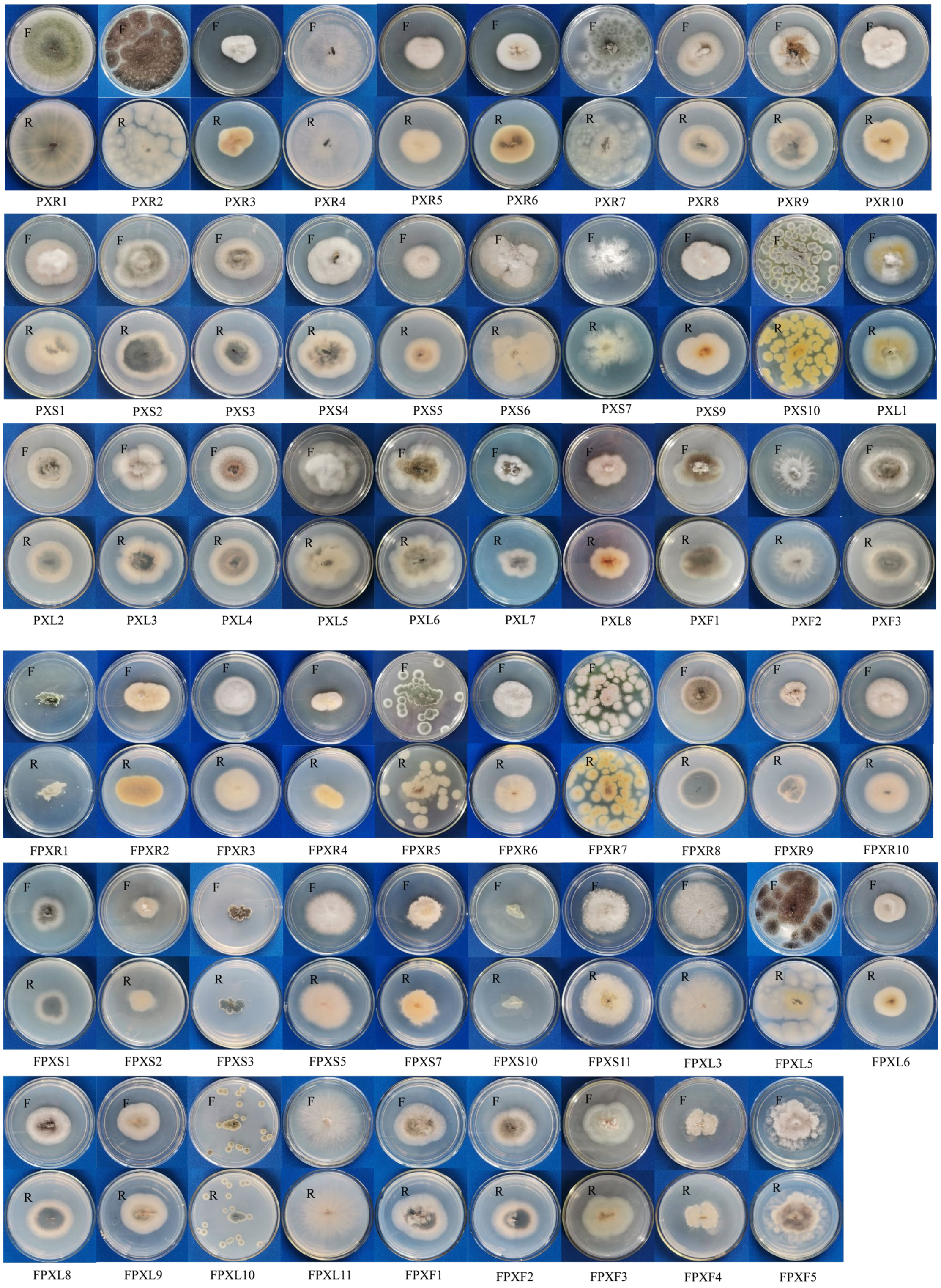

Figure S5. Colonies of some endophytic fungi isolated from *C. pareira* on PDA cultured for 6 days. F: Front view of culture on PDA. R: Reverse view of culture on PDA.
