# Supplementary material for: Community Structure Diversity of Endophytic Fungi in Cissampelos pareira from Different Habitats and Their α-Glucosidase Inhibitory Activity
Source: J Fungi (Basel). 2025 Aug 22;11(9):615. doi: 10.3390/jof11090615 (PMC12470284; doi:10.3390/jof11090615)
Supplement: Supplementary file 1 [file jof-11-00615-s001.zip › Figure S6 Fungi community composition at the family level.pdf]

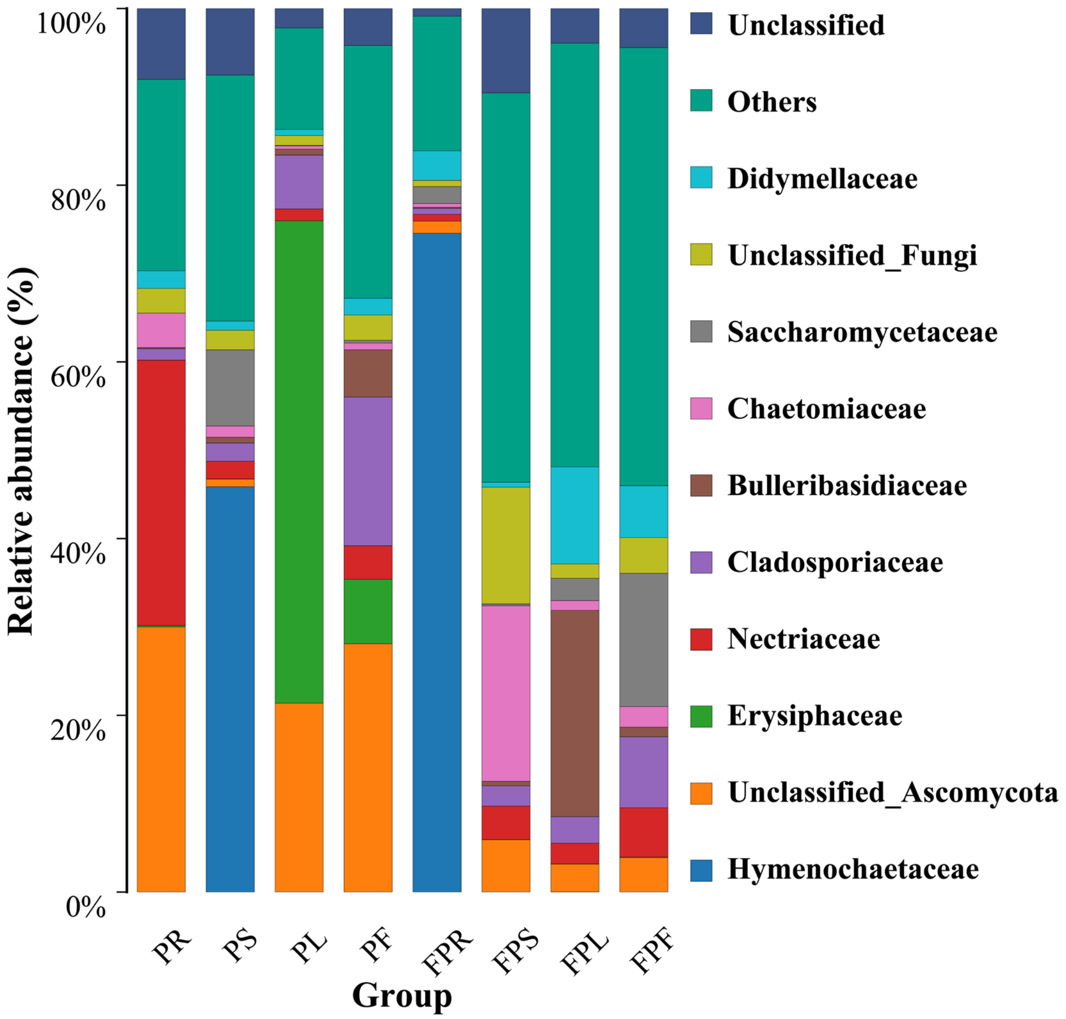

Figure S6. Fungi community composition at the family level. PR: potted *C. pareira* root, PS: potted *C. pareira* stem, PL: potted *C. pareira* leaf, PF: potted *C. pareira* flower, FPR: non-potted *C. pareira* root, FPS: non-potted *C. pareira* stem, FPL: non-potted *C. pareira* leaf, FPF: non-potted *C. pareira* flower.
