# Supplementary figures and images for: Community Structure Diversity of Endophytic Fungi in Cissampelos pareira from Different Habitats and Their α-Glucosidase Inhibitory Activity
Source: J Fungi (Basel). 2025 Aug 22;11(9):615. doi: 10.3390/jof11090615 (PMC12470284; doi:10.3390/jof11090615)

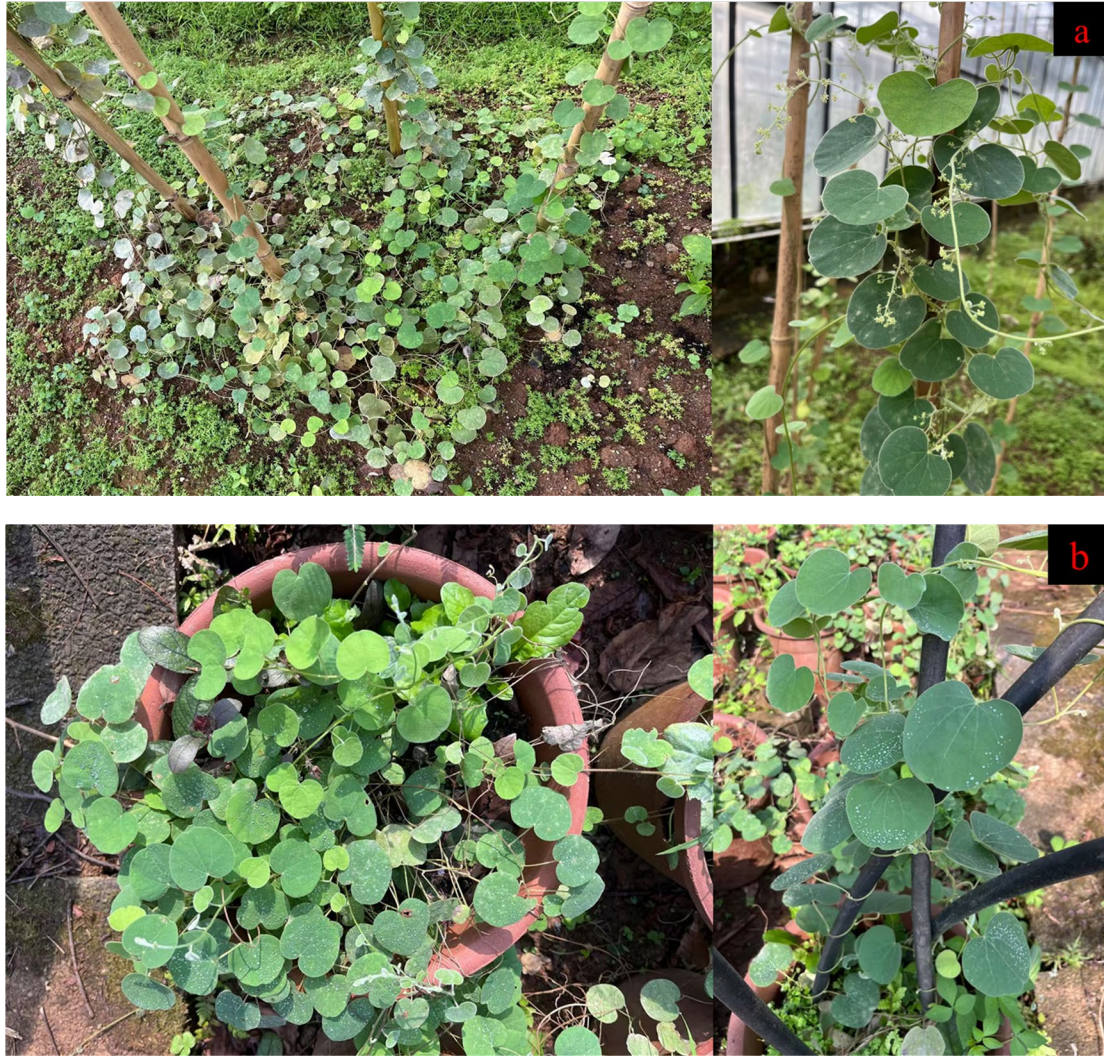

**Figure S1.** *C. pareira* in two distinct habitats. (a) Non-potted *C. pareira*; (b) Potted *C. pareira*

Supplement: Supplementary file 1 [file jof-11-00615-s001.zip › Figure S1 C. pareira in two distinct habitats.pdf]

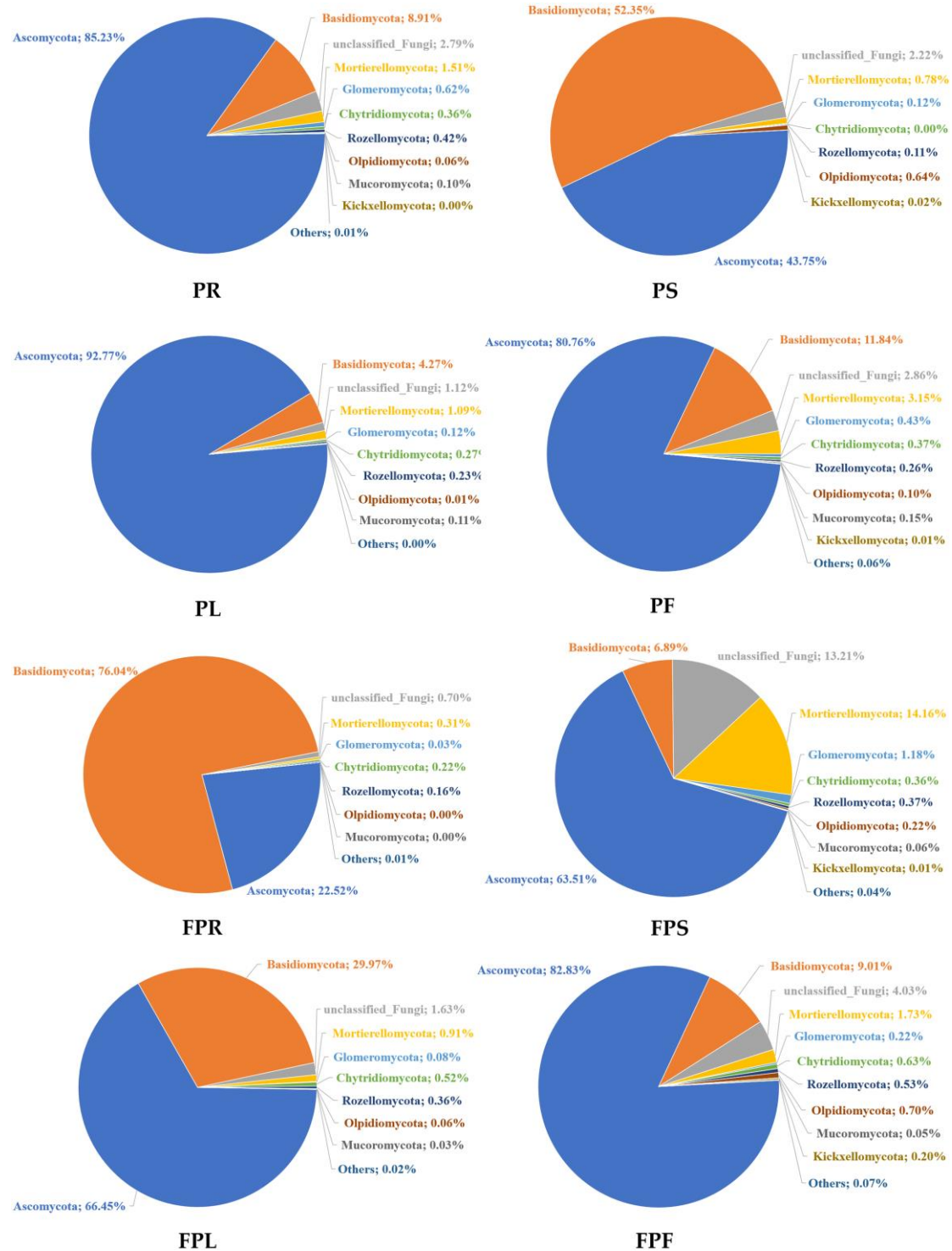

**Figure S3.** Community analysis of endophytic fungi from *C. pareira* at the phylum level.

Supplement: Supplementary file 1 [file jof-11-00615-s001.zip › Figure S3 Community analysis of endophytic fungi from C. pareira at the phylum level.pdf]

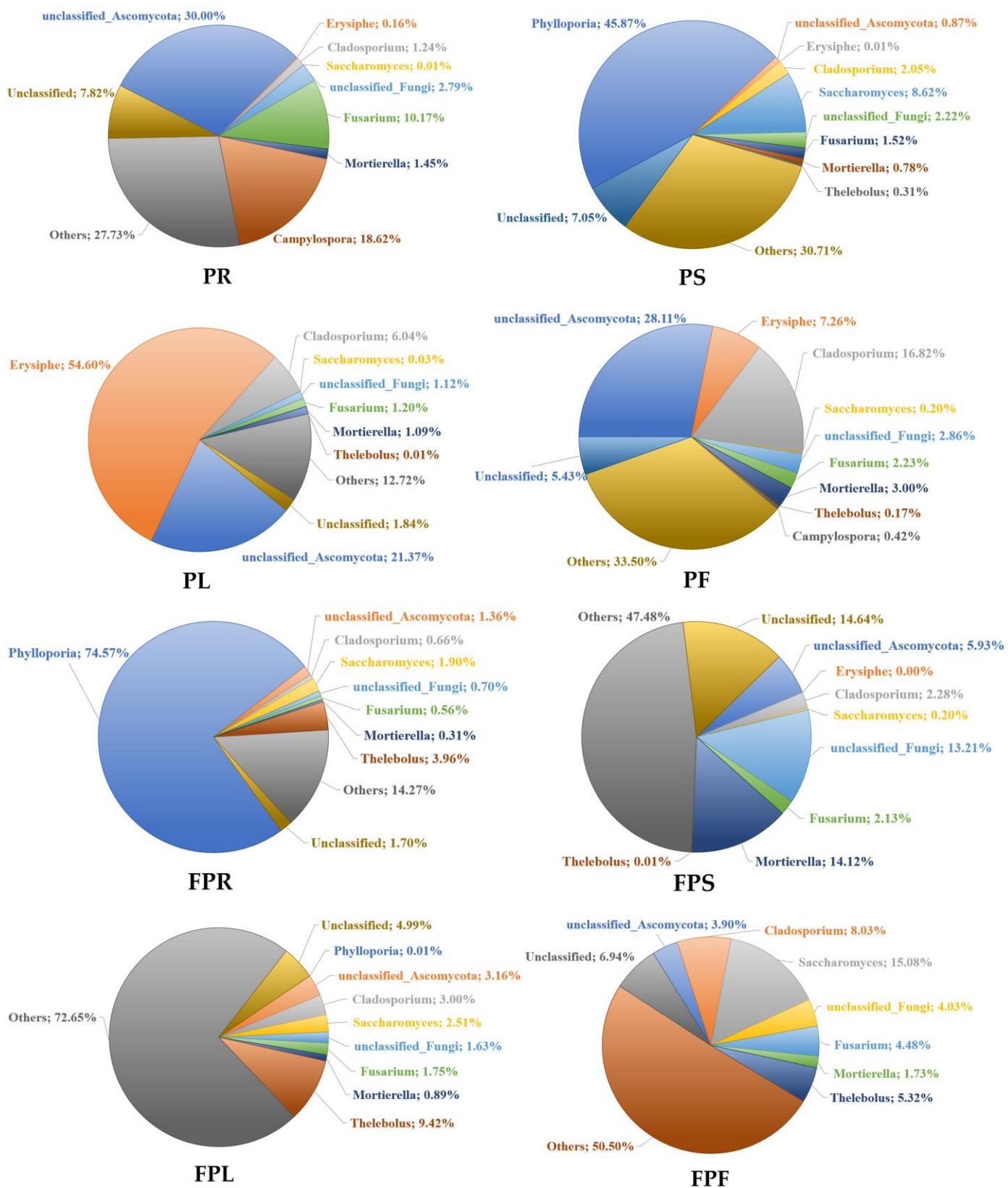

**Figure S4.** Community analysis of endophytic fungi from *C. pareira* at the genus level.

Supplement: Supplementary file 1 [file jof-11-00615-s001.zip › Figure S4 Community analysis of endophytic fungi from C. pareira at the genus level.pdf]
